# Anti-NMDA receptor encephalitis and overlapping demyelinating disorder in a 20-year old female with borderline personality disorder: proposal of a diagnostic and therapeutic algorithm for autoimmune encephalitis in psychiatric patients “case report”

**DOI:** 10.1186/s12888-021-03269-0

**Published:** 2021-07-15

**Authors:** David Weiss, Lisa Kertzscher, Magdalena Degering, David Wozniak, Michael Kluge

**Affiliations:** 1 Department of Psychiatry and Psychotherapy, University of Leipzig, Leipzig, Germany; 2 Department of Neurology, Rostock University Medical Center, Germany; Gehlsheimer Straße 20, 18147 Rostock, Germany

**Keywords:** Anti-NMDA receptor encephalitis, Neuropsychiatric red flags, Demyelinating overlap syndrome, Diagnostic and therapeutic algorithm, Case report

## Abstract

**Background:**

Anti-NMDA receptor encephalitis (NMDAR-E) is an autoimmune encephalitis (AE) mainly affecting young females. It typically presents with isolated psychiatric symptoms (e.g. depressed mood) at first and neurological abnormalities (e.g. seizures, movement disorders) only develop later. Thus, there is a high risk of overlooking NMDAR-E in patients with preexisting psychiatric illness due to symptom overlap in the prodromal period of the disease when treatment is most effective. Although rare, concomitant or sequential development of a demyelinating disorder is increasingly recognized as an associated disease entity (overlap syndrome), with immediate diagnostic and therapeutic implications.

**Case presentation:**

We report a patient with a borderline personality disorder (BPD), which developed NMDAR-E and an overlapping demyelinating disorder with anti-Myelin oligodendrocyte glycoprotein (MOG) -IgG positivity. The initial clinical presentation with predominantly affective symptoms (e.g. mood lability, anxiety, depressed mood) lead us to suspect an exacerbation of the BPD at first. However, acute changes in premorbid behavior, newly developed psychotic symptoms and memory deficits lead us to the correct diagnosis of an AE, which was further complicated by the development of a demyelinating disorder. As a result of impaired illness awareness and psychosis, diagnostic and treatment was difficult to carry out. The symptoms completely remitted after treatment with methylprednisolone 1 g daily for 5 days and 5 cycles of plasma exchange.

**Conclusions:**

Continuous awareness for neuropsychiatric clinical warning signs in patients with a pre-diagnosed psychiatric disorder is important for a timely diagnosis. Therefore, we believe that the diagnostic and therapeutic algorithm provided here, for the first time specifically addressing patients with preexisting psychiatric illness and integrating overlap syndromes, can be a useful tool. Moreover, in order to timely perform diagnostics and treatment, judicial approval should be obtained rapidly.

## Background

Anti-NMDAR receptor encephalitis (NMDAR-E) is a well-defined immune-mediated disease, based on the presence of antibodies against the NR1 subunit of the N-methyl-D-aspartate (NMDA) receptor [1]. Clinically, NMDAR-E is characterized by a multistage progression of symptoms: prodromal symptoms (e.g. headache, mood changes), psychotic features (e.g. delusions), cognitive decline (e.g. working memory), epileptic seizures, movement disorders (e.g. dyskinesias, dystonic body posture), autonomous instability and reduced consciousness [2]. Diagnosis can be difficult in patients with preexisting psychiatric illnesses due to symptom overlap in the prodromal period, when differentiating neurological symptoms are still lacking [3]. NMDAR-E can coexist with an overlapping demyelinating syndrome, including Myelin oligodendrocyte glycoprotein (MOG) antibody disease [4], implicating the need for additional diagnostics and different therapeutic approaches [5].

## Case presentation

Ms. A. is a 21-year old woman with a 9-year history of borderline personality disorder (BPD) symptoms, with BPD being diagnosed aged 19 when being treated for this reason for the first time. BPD was characterized by experience of emptiness, self-harming behavior (cutting), including three suicide attempts aged 15 and 16, and abuse of illicit drugs, including amphetamines, cannabis and LSD. The father was described as “aggressive”, but had no formal psychiatric diagnosis or treatment. Otherwise, the family history and the further patient’s history were unremarkable. On admission, BPD was reasonably well controlled, with no hospitalizations or self-harming behavior in the 12 months before admission, a stable partnership, an apprenticeship and a reduced drug abuse limited to sporadic cannabis consumption. Ms. A. occasionally took over-the-counter hypnotics (e.g. valerian), but no other psychotropic medication.

Two weeks before admission, Ms. A. developed persistent headache. On admission, the patient presented with poor attention, anxiety and depressed mood. One week prior to admission, her school was shut down due to the coronavirus pandemic, causing her a lot of distress and fear for the future. In a text message to her father she wrote: “I have no idea how things will develop; I am afraid of getting a nervous breakdown”. The day after, she showed signs of confusion for the first time, while still being rational: “I am losing it, everything feels weird, I should see a physician”. On the same day she experienced a “nervous breakdown” during her work in a hardware store. Three days later, she was emergently admitted to our clinic, showing mood lability, anxiety and depressed mood. These symptoms were initially interpreted as stress-related exacerbation of the BPD. Neurological and physical examinations were unremarkable.

**Fig. 1:**
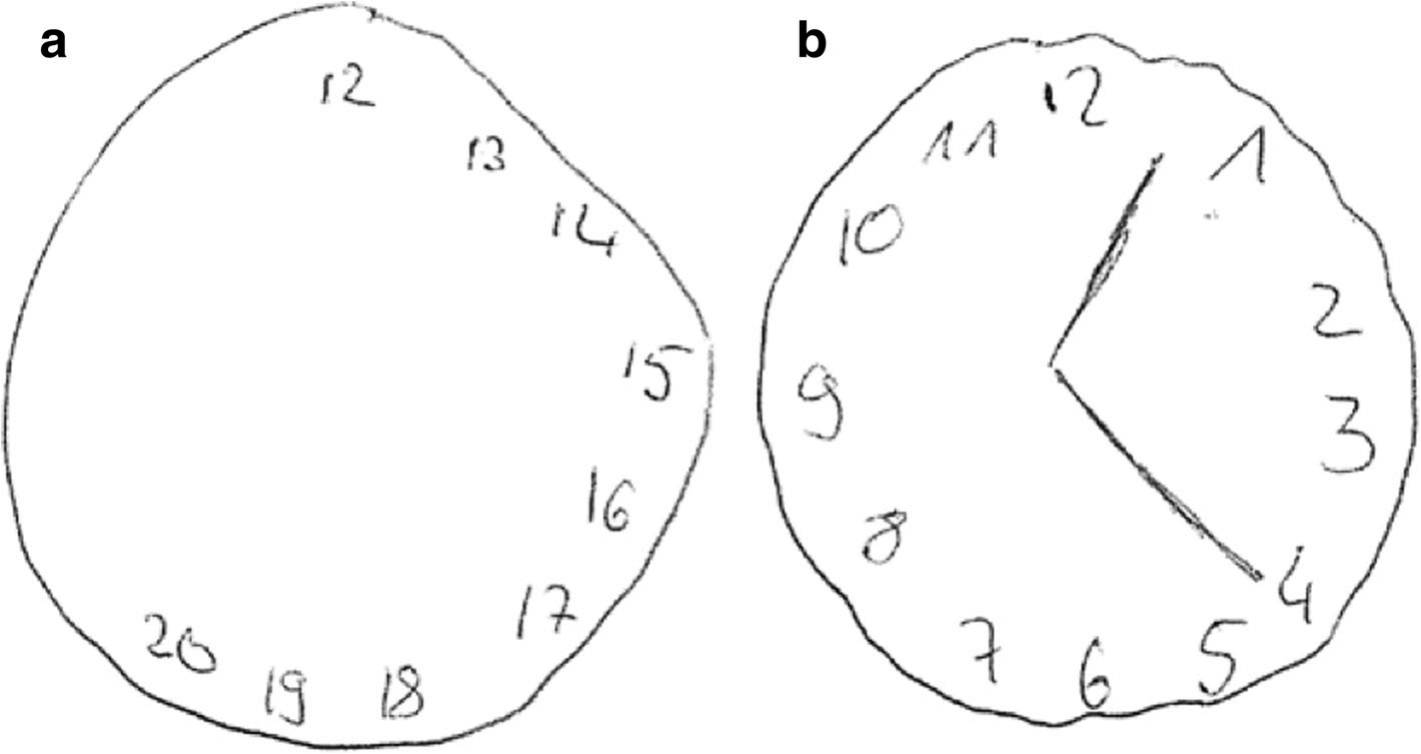
**a** Initial clock-drawing test before therapy. **b** Clock-drawing test at the day of discharge

One week after admission, the patient’s cognitive functions deteriorated markedly, with concentration loss, deficits in working memory and disorientation. The Montreal Cognitive Assessment (MoCA) [6] sum score was 18/30, showing significant deficits in delayed recall, abstraction, serial subtraction, verbal fluency and visuospatial-executive functions. The clock-drawing test was grossly inaccurate (Fig. 1a). In addition, the patient developed psychotic symptoms with paranoid suspiciousness and distrust of others, claiming that the ward staff was made up of “actors”, and bizarre thoughts, like asking why she was born. Therefore, olanzapine 20 mg daily was started, which was well tolerated, but had no significant effect. Furthermore, Ms. A. reported having recurring night terrors and suicidal thoughts. The neurological and physical examinations remained unremarkable.

Given the subacute onset, the patient’s young age, gender and psychopathology with a newly developed mixed mood-psychosis syndrome [7] and headache, we considered an organic cause, such as AE. Therefore, we planned to perform a diagnostic work-up, including brain MRI, EEG, serum screening for autoantibodies associated with AE and lumbar puncture. However, Ms. A. refused all diagnostics and treatment except for blood drawing, having anosognosia with poor insight into the presence and nature of the illness. When being confronted with her poor performance in the clock-drawing test, she replied: “I have been this way for all my life, I have never been able to draw a clock; what is this all about, I want to leave the hospital”.

In line with our hypothesis, we found elevated NMDAR-antibodies (antibody-titer 1:32) in the serum, indicating an NMDAR-E. Also, the antibody-titer of previously normal antinuclear antibodies (ANA) was elevated (1:160), indicative of a newly developed systemic autoimmune disease. Stressing the severity of the illness and its usually harmful course, we successfully applied for an involuntary treatment with coercive disease-specific diagnostics and treatment.

**Fig. 2:**
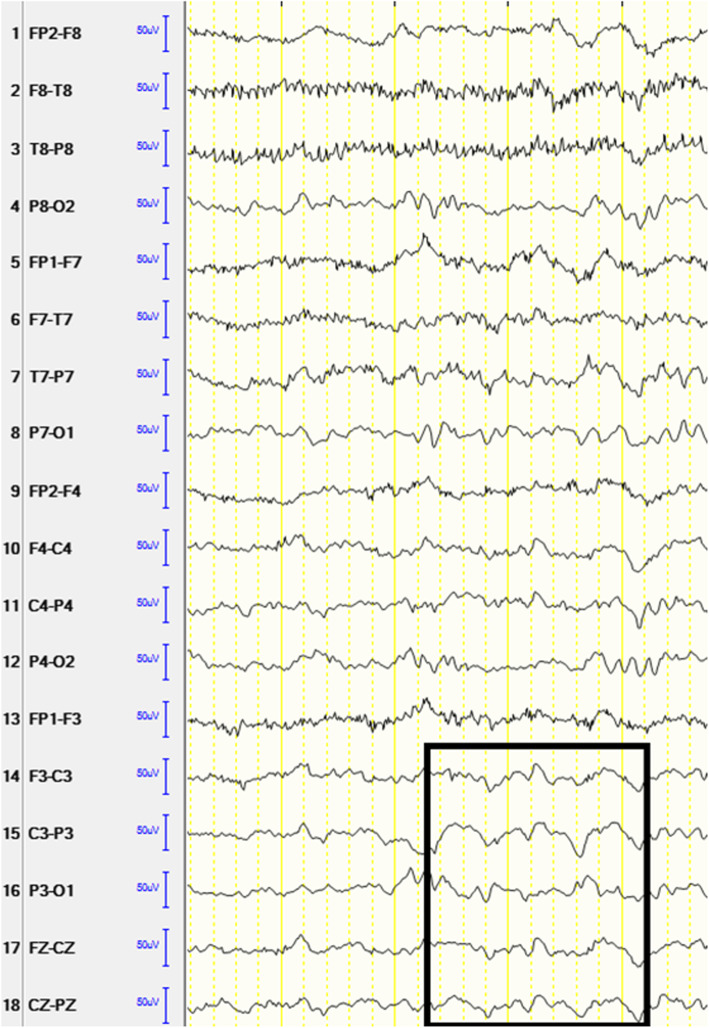
EEG showing diffuse theta and delta slowing

The brain MRI was only feasible under anesthesia. Upon first evaluation, the MRI was considered unremarkable, especially without pathological findings suggestive of an AE, like abnormal signals in the mesial temporal lobes or white matter lesions. The EEG displayed diffuse theta and delta slowing (Fig. 2). The cerebrospinal fluid (CSF) showed several abnormal findings: a monocytic pleocytosis (122 cells per mm3), CSF-specific oligoclonal bands (OCB, Type 2), indicating intrathecal immunoglobulin (antibody) production, and increased CSF/serum antibody indices for rubella and varicella zoster virus (incomplete MRZ reaction). CSF concentrations of protein, lactate and glucose were normal. The rather high CSF pleocytosis - usually autoimmune encephalitis is accompanied by mild pleocytosis from 5 to 100 per mm3 [2]- prompted us to consider infectious causes (especially a preceding or concurrent herpes simplex encephalitis (HSV), being a well-known trigger for NMDAR-E [8]). Since especially HSV encephalitis, but also bacterial meningoencephalitis, can take an aggressive course, we immediately started treatment with acyclovir, cefotaxime and ampicillin. After the CSF PCR (polymerase chain reaction) test results for Herpesviridae (HSV 1, VZV, CMV, EBV, FSME, HIV, JCV, SARS-CoV-2), bacterial and fungal etiologies came back negative the next day, we started intravenous immunosuppressive therapy with methylprednisolone1g daily for 5 days. NMDA antibody titer in the CSF was 1:10, confirming the diagnosis of NMDAR-E.

**Fig. 3:**
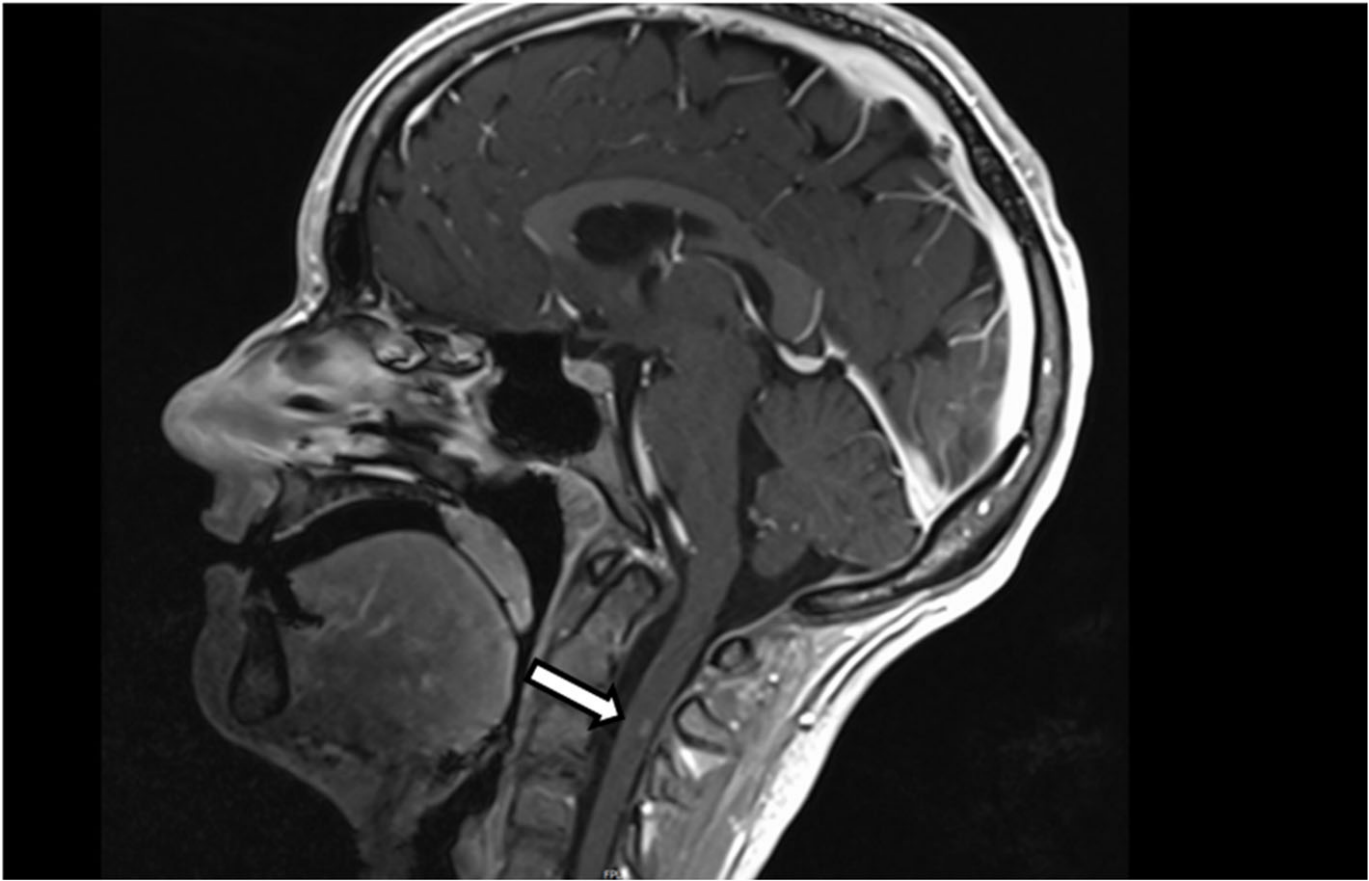
T1 weighted image (sagittal), showing contrast enhancement of the lesion in the dorsal column of the cervical spinal cord C2/C3

**Fig. 4:**
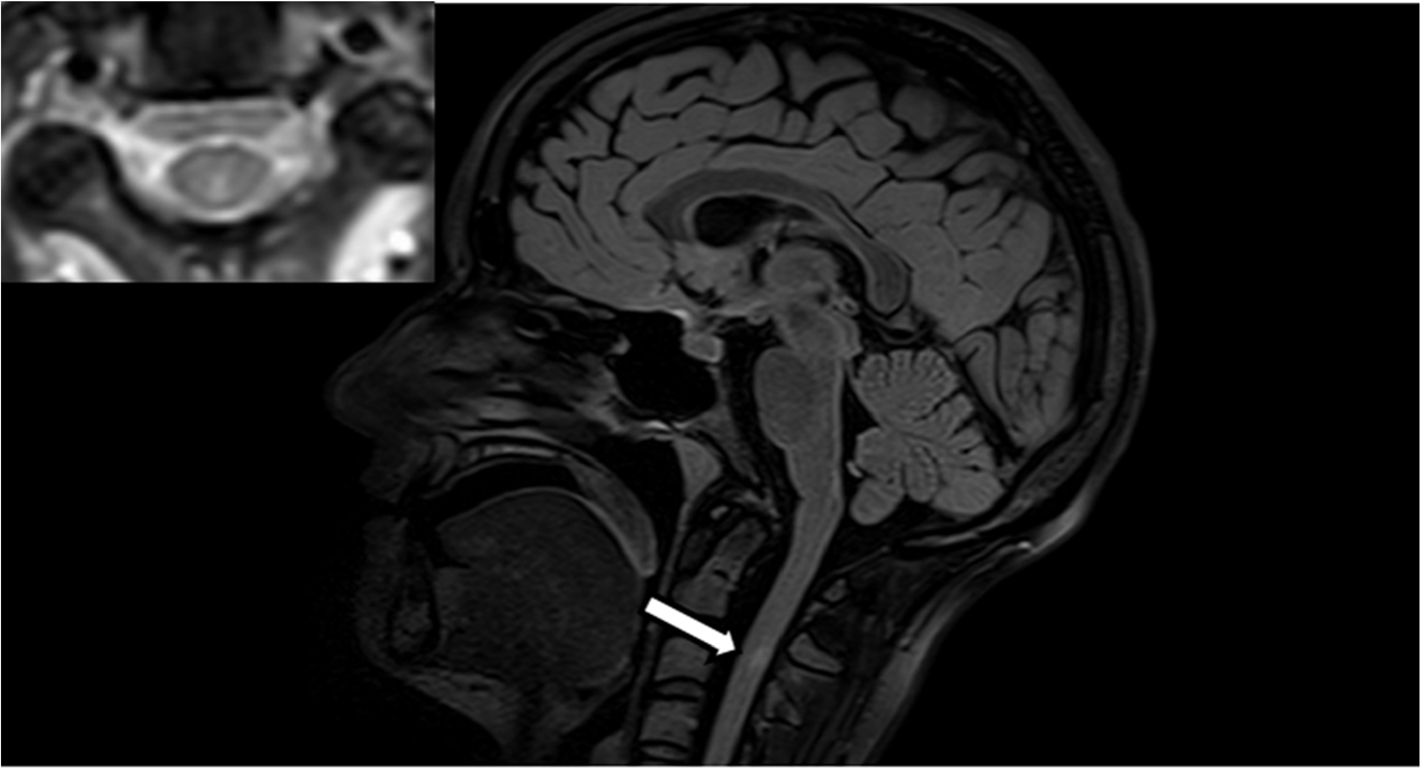
MRI image (FLAIR sagittal and T2 transversal plane), showing the lesion in the dorsal column of the cervical spinal cord C2/C3

As a result, cognitive performance improved, and psychotic symptoms partly remitted within 5 days. However, neuropsychological testing still demonstrated significant memory deficits and thinking was still disorganized. Moreover, Ms. A. experienced 2 presyncope’s and developed sinus tachycardia (up to 150 bpm), suggestive of autonomic instability, which is a common feature of NMDAR-E [4]. Then, 3 days after discontinuation of high dose glucocorticoid therapy, Ms. A. experienced transient blurred vision on both eyes and unsteady gait. Hence, the brain MRI was re-evaluated for demyelination signs. In fact, an enhancing demyelinating Multiple sclerosis (MS)-like lesion now was detected in the dorsal column of the cervical spinal cord C2/C3 (Figs. 3 and 4). Assuming a newly acquired demyelinating disorder with optic nerve involvement and afferent gait disturbance, we tested for MOG-IgG and Aquaporin 4 (AQP4) antibodies in serum and CSF, being both relevant mediators in autoimmune demyelinating syndromes of the CNS [9]. MOG-IgG antibodies came back positive (antibody titer 1:10) in serum, but not in the CSF; AQP4 antibodies were negative, both in serum and CSF.

Because of the insufficient response to glucocorticoid treatment, the therapy was escalated: Ms. A. underwent 5 cycles of plasma exchange, while oral prednisone 60 mg daily was continued. Already after the first cycle, cognitive capabilities improved, especially working memory capacity and visuospatial-executive functions. The MoCA sum score improved to 25/30. Also, the clock drawing test substantially improved (Fig. 1b). Blurred vision and unsteady gait remitted. Correspondingly, a control MRI showed disappearance of the previously enhancing demyelinating lesion in the cervical spinal cord. In addition, we performed evoked potentials (VEP, MEP, SEP) that were unremarkable (no significant differences in latency and amplitude).

In the meantime, an ovarian teratoma, being a paraneoplastic cause for NMDAR-E in approximately 50% of female patients [10], had been ruled out by pelvic MRI. Other occult malignancies were ruled out by whole body PET/CT. Ms. A. was discharged substantially improved, with only subtle cognitive impairments, which fully remitted upon neuropsychological re-evaluation 4 weeks later. Antipsychotic medication with olanzapine 10 mg daily and immunosuppressive therapy with prednisone 60 mg daily (with a tapering schedule) was continued, since patients with anti-NMDA receptor encephalitis are at risk for relapse [5]. Follow-up neuropsychological testing and monitoring of therapy adherence is ensured by our outpatient clinic.

## Discussion and conclusions

**Fig. 5:**
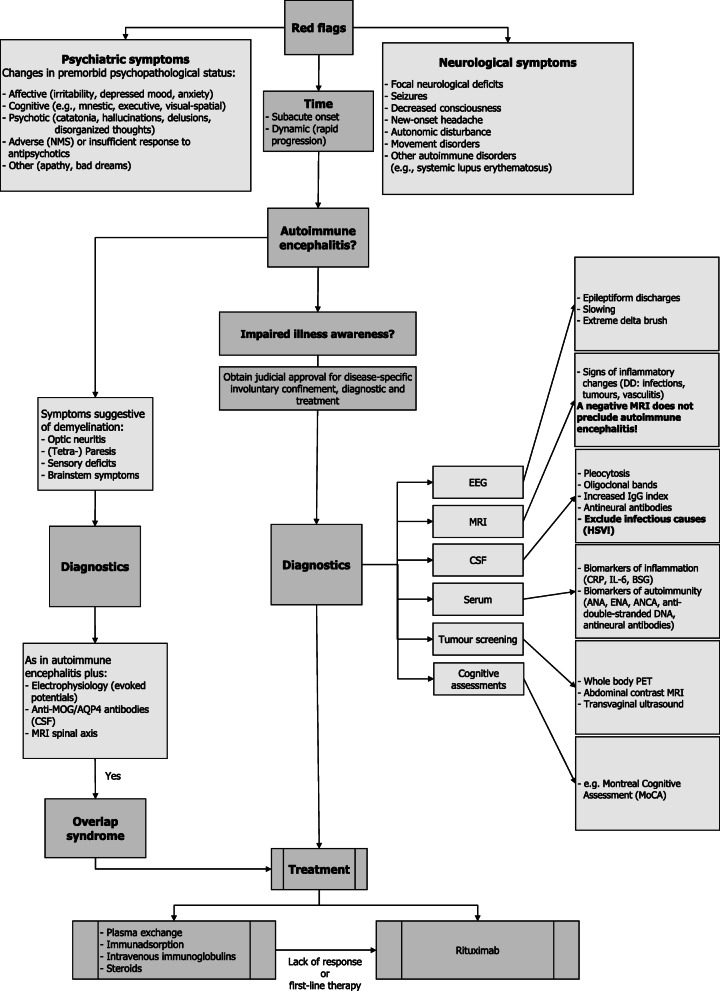
Diagnostic and therapeutic algorithm, specifically considering patients with preexisting psychiatric illness and integrating overlap syndrome (based on [2, 4, 7, 8, 11–15]

Herein we report a patient with a preexisting BPD, which developed NMDAR-E and an overlapping demyelinating disorder. There are several important conclusions to draw from this case:

Firstly, diagnosing AE in already psychiatrically ill patients is difficult. A high level of alertness is needed, particularly for early diagnosis, when differentiating neurological features are still lacking. Thus, we suggest considering the psychiatric red flags in our proposed algorithm (Fig. 5) as a guide for earlier diagnosis. The difficulty arises primarily because symptoms of autoimmune encephalitis may mimic symptoms of the preexisting psychiatric condition on the one hand, and because a further cause for psychiatric symptoms is simply not being expected, on the other hand [2].. As a result, an autoimmune encephalitis can be easily overlooked, at least at the beginning [16]. In our case, the patient’s initial symptoms, mostly affecting mood, were interpreted as an exacerbation of her preexisting BPD.

Secondly, in patients with symptoms atypical for AE (e.g. paresis), demyelinating disorders should be considered (Fig. 5). Demyelinating disorders such as anti-MOG disease (MOG-AD), can develop concomitantly or sequentially in individuals with NMDAR-E [4, 17]. Likewise, patients with a history of demyelinating disorder should be tested for co-existing NMDAR-E, if presenting with atypical symptoms such as psychosis or seizures [17]. Although MOG-IgG antibodies were tested positive in our patients serum, some caution should be exerted in diagnosing MOG-AD [18], since the CSF profile of our patient is more suggestive of an MS-like immune response, possibly elicited by NMDAR antibodies: positive MRZ reaction, positive OCBs, preserved blood-CSF barrier integrity and normal CSF albumin concentration [19]. Since oligodendrocytes contain NMDAR, a direct immuno-pathophysiological demyelinating effect of NMDAR-antibodies is conceivable [4]; thus MOG antibodies may rather represent a bystander effect or epiphenomenon in our case. Since the criterion of dissemination in space for diagnosing MS isn’t fulfilled and given the absence of any history or clinical signs of MS before the onset of encephalitis, pre-existing MS and now superimposed NMDAR-E seems to be unlikely. Longitudinal reevaluation in this regard however is important, since there are rare case reports of co-existent MS and NMDAR-E [20, 21]. Of note, the unremarkable evoked potentials were performed after having administered 2 cycles of plasma exchange, at a time when the patients’ symptoms already had remitted.

Thirdly, as a result of the impaired illness awareness and often psychosis, patients with AE are frequently unable to consent and commonly refuse diagnostics, significantly delaying urgent treatment. Hence, it is very important to rapidly obtain judicial approval for involuntary confinement, diagnostics and treatment, ultimately to restore the patient’s autonomy. Since diagnostics and treatment of AE is invasive (e.g. lumbar puncture, plasma exchange), explicit approval for disease-specific diagnostics and treatment should be obtained [22].

AE is a comparatively new disease entity [16]. Therefore, randomized controlled trials are still lacking and thus treatment approaches are largely based on considerable clinical experience [23]. While there are already profound algorithms for diagnostics and treatment of AE [5, 8, 11, 22, 23], we believe that the algorithm provided here, specifically addressing patients with preexisting psychiatric illness and integrating overlap syndrome, can be an additional useful tool for a timely diagnosis and rational treatment. Although methylprednisolone, plasma exchange and intravenous immunoglobulin are considered as first-line therapy, we, in line with others [5, 23], propose B-cell depletion with rituximab as first-line and maintenance therapy, since it is associated with fewer relapses and better outcomes [23, 24]. Especially patients with a demyelinating overlap syndrome (and MOG antibody positivity) could benefit, given the shared antibody-mediated immunopathology. In our case, insurance coverage for rituximab as first-line and maintenance therapy (off-label use) was denied. However, rituximab (typically given every 6 months) seems to be especially advantageous as maintenance therapy in psychiatric patients, since lack of adherence is frequent in psychiatric patients [25].

In conclusion, the complex case presented here describes different diagnostic and therapeutic problems encountered with AE, specifically NMDAR-E, and aims at providing specific proposals, based on comprehensive literature review, for their solution. However more clinical experience and long-term reevaluation is needed.

## Data Availability

All data and material supporting our findings are contained within the manuscript.

## Abbreviations

AE: Autoimmune encephalitis
ANA: Antinuclear antibodies
AQP4: Aquaporin 4
BPD: Borderline personality disorder
CMV: Cytomegalovirus
CSF: Cerebrospinal fluid
EBV: Epstein-Barr virus
EEG: Electroencephalogram
FSME: Tick-borne encephalitis virus infections
HIV: Human immunodeficiency virus
HSV: Herpes simplex virus
JCV: John Cunningham virus
MEP: Motor evoked potentials
MOG-AD: Anti-MOG disease
MOG: Myelin oligodendrocyte glycoprotein
MoCA: Montreal Cognitive Assessment
MRI: Magnetic resonance imaging
MS: Multiple sclerosis
NMDAR-E: Anti-NMDA receptor encephalitis
NMDA: N-methyl-D-aspartate
OCB: Oligoclonal bands
SARS-CoV-2: Severe acute respiratory syndrome coronavirus 2
SEP: Somatosensory evoked potential
VEP: Visual evoked potentials
VZV: Varicella zoster virus
